# Editorial: Multi-omics: Trends and applications in clinical research

**DOI:** 10.3389/fmolb.2022.994239

**Published:** 2022-11-24

**Authors:** Alessandra Sussulini, Jianguo Xia, Matej Orešič

**Affiliations:** ^1^ State University of Campinas, Campinas, Brazil; ^2^ McGill University, Montreal, QC, Canada; ^3^ Örebro University, Örebro, Sweden

**Keywords:** multi-omics, metabolomics, proteomics, data integration, transcriptomics

Omics-based studies have substantially contributed to the emergence and progress of systems biology over the past decades. Each layer of omics data provides a unique perspective on the biological system at a particular molecular level (e.g., RNA, proteins, metabolites), with all levels being involved in different yet inter-related biological processes. However, data at any individual omics level cannot fully explain how these different multi-layered biological processes interact and how they lead to emergence of complex phenotypes. To answer these questions, multi-omics strategies have been increasingly employed to obtain a broader view of the interactions that connect genotypes to phenotypes of interest. Omics data integration can provide a more reliable and holistic picture of the biochemistry and dynamics of biological systems, as compared to data from any omics layer alone. Multi-omics is also a powerful tool for discovery and prioritization of biomarkers, which is a topic of high interest in clinical research. To understand biological processes within the systems biology context, various statistical and computational tools are typically employed in multi-omics studies. In clinical studies, data integration across different omics is a promising tool for the early detection of various diseases, as well as for evaluating the efficacy of different treatments. Therefore, the aim of this Research Topic was to cover advances in the multi-omics research field with consideration of clinical applications. This Research Topic of five articles involves multi-omics studies of different diseases.

By integrating genomics and transcriptomics data, Wang et al. evaluated the effect of smoking history on cancer patients and proposed a 46-gene model to predict overall survival of the patients, as well as to disease-specific survival and progression-free intervals.

To understand esophageal squamous cell carcinoma (ESCC), Zang et al. combined comprehensive metabolomic characterization with expression profiling of interleukin enhancer binding factor 2 (ILF2) and ILF3, proteins known to contribute to the occurrence and development of several types of malignancy. The results indicated that a series of acyl-carnitines were positively correlated with the levels of ILF2 in ESCC tissues and that elevated ILF3 expression in ESCC tissues was negatively correlated with the levels of several key intermediate metabolites in the glycolysis pathway.

The development of malignant gliomas and its relation to lysine acetylation regulators (LARs) were investigated by Tu et al. through RNA-seq transcriptome data and microarray mRNA expression profile correlated to clinicopathological features. The authors identified three glioma subtypes (LA1, 2, and 3), and proposed a LAR-signature related to tumor mutation burden of the patients which presented strong and independent prognostic value for glioma patients in both training and validation datasets.

Cerebral infarction (CI) was studied by Chen et al. by performing an untargeted metabolomics approach of serum samples of patients across different CI phases. Metabolic pathway analysis indicated that phenylalanine metabolism was involved in the development of CI within 3–7 days and galactose metabolism, inositol phosphate metabolism, and ascorbate and aldarate metabolism were differentially active only between the CI diabetic patients and healthy control groups.

Finally, Qin et al. studied right ventricular failure (RVF) in a monocrotaline (MCT)-induced PAH rat model. Proteomic and metabolomic profiles of the RV myocardium were obtained and bioinformatics analysis indicated that elevated intracellular calcium concentrations and inflammation might contribute to myocardial proliferation and contraction, which may be beneficial for maintaining the compensated state of the RV. Ferroptosis, mitochondrial metabolic shift, and insulin resistance are significantly involved in the RVF stage. Dysregulated iron homeostasis, glutathione metabolism, and lipid peroxidation related to ferroptosis may contribute to RV decompensation.

Most current literature on multi-omics studies comprises the integration of genetic and transcriptomics data. Nevertheless, there is a growing interest in the integration of proteomics and metabolomics with the aim to provide biological information closer to the phenotype, as approached in this Research Topic.

